# Big Data and Network Biology 2016

**DOI:** 10.1155/2017/9432460

**Published:** 2017-01-26

**Authors:** Shigehiko Kanaya, Md. Altaf-Ul-Amin, Samuel K. Kiboi, Farit Mochamad Afendi

**Affiliations:** ^1^Nara Institute of Science and Technology (NAIST), Nara 630-0192, Japan; ^2^University of Nairobi, Nairobi 00100, Kenya; ^3^Bogor Agricultural University, West Java 16680, Indonesia

Big data science towards biology has emerged as an important research and application field. The scope of big data bioinformatics and the need for integrated systems biology applications have posed significant challenges to computing systems. With the growing deluge of molecular biological data, big data methodologies, including data mining algorithms and processing techniques, are becoming particularly relevant. These encompass data accumulation, storage, retrieval, classification, and visualization. These have become increasingly important themes of research in the past decade. Network analysis has become popular for systems level analysis of biological phenomena. Both micro- and macrolevel biological networks of various types facilitate the development of theory and methodology for solving core scientific issues. The application ranges from studies of ecological structure, biodiversity and environment, and evolution and extinction of species. It is also more widely used in studies on metabolic regulation and biomarker identification particularly for noncommunicable diseases such as cancer, Alzheimer's disease, and diabetes. The versatility of these topics, theory and methodology of big data and network biology, justifies the aims and scope of this special issues. The present special issue is however not an exhaustive representation of these topics.

This special issue contains six papers. One article presents a biomedical text mining approach and another article talks about algorithms for inferring gene regulatory networks. Four other papers present methodology related to genomics, transcriptomics, metabolomics, and drug repositioning.

The paper “Novel Approach to Classify Plants Based on Metabolite-Content Similarity” proposed an unsupervised approach to classify plants based on their known metabolite content data. Plants were classified based on structurally similar metabolite groups to reduce the influence of incomplete data. The resulting plant clusters were found to be consistent with known evolutional relations of plants and reveal the significance of metabolite content as a taxonomic marker.

The paper “A Systematic Framework for Drug Repositioning from Integrated Omics and Drug Phenotype Profiles Using Pathway-Drug Network” proposes a systematic framework that employs experimental genomic knowledge and pharmaceutical knowledge to reposition drugs for a specific disease. The experimental results showed that the proposed framework is a useful approach to discover promising candidates for breast cancer treatment.

The paper “MapReduce Algorithms for Inferring Gene Regulatory Networks from Time-Series Microarray Data Using an Information-Theoretic Approach” proposes new MapReduce algorithms for inferring gene regulatory networks (GRNs) on a Hadoop cluster in a cloud environment. These algorithms employ an information-theoretic approach to infer GRNs using time-series gene expression profiles. Experimental results show that their MapReduce program is much faster and achieves slightly better prediction accuracy than a state-of-the-art R program.

The paper “Correlation-Based Network Generation, Visualization, and Analysis as a Powerful Tool in Biological Studies: A Case Study in Cancer Cell Metabolism” introduces one of a series of methods for correlation-based network generation and analysis using freely available software. The pipeline used published metabolomics data of a population of human breast carcinoma cell lines MDA-MB-231 under normal and hypoxia conditions. The analysis revealed significant differences between the metabolic networks in response to the tested conditions.

The paper “Semisupervised Learning Based Disease-Symptom and Symptom-Therapeutic Substance Relation Extraction from Biomedical Literature” presents a method of constructing two models for extracting the relations between the disease and symptom, and symptom and therapeutic substance from biomedical texts, respectively. The authors apply two semisupervised learning algorithms, Co-Training and Tri-Training, to boost the relation extraction performance.

Horizontal gene transfer (HGT) has had an important role in eukaryotic genome evolution. The paper “Horizontally Transferred Genetic Elements in the Tsetse Fly Genome: An Alignment-Free Clustering Approach Using Batch Learning Self-Organising Map (BLSOM)” employs BLSOM to explore the genome of* Glossina morsitans* for evidence of HGT from microorganisms. The predicted donors of HGT candidate include diverse bacteria that have not previously been associated with the tsetse fly. These findings provide a basis for understanding the coevolutionary history of the tsetse fly and its microbes and establish the effectiveness of BLSOM for the detection of HGT.

